# Laennec's approach for central liver resection of colorectal cancer liver metastasis adjacent to the hepatic hilum that occurred 10 years after colectomy: A case report

**DOI:** 10.1016/j.ijscr.2024.109327

**Published:** 2024-02-01

**Authors:** Anton Burlaka, Veronika Rozhkova, Romanna Pavliuk, Oleksandr Chukanov, Andriy Beznosenko

**Affiliations:** a Department of thoraco-abdominal oncology, National Cancer Institute, Kyiv, Ukraine; b Lifescan diagnostic center, Kyiv, Ukraine

**Keywords:** Colorectal cancer liver metastasis, Late metachronous metastasis, Liver resection, Laennec's capsule, Case report

## Abstract

**Introduction and importance:**

Colorectal cancer ranks as one of the most common cancer globally. About half of the patients experience a disease recurrence in the form of сolorectal cancer liver metastasis (CRLM) within the first 5 years of the course of the disease. However, there are rare cases of delayed onset of liver metastasis, that occur after cessation of standard follow-up.

**Case presentation:**

A 38-year-old woman was referred to our institute with a metastatic liver mass adjacent to the liver hilum. The patient had sigmoid colectomy 10 years ago. After 6 cycles of chemotherapy, she underwent a central liver resection: Sg4, Sg5, Sg8v + middle hepatic vein with surgical skeletonization of 1st and 2nd order hepatic pedicles. The patient was discharged on 5th post-operative day. After 18th month of follow-up, the patient was alive without any signs of recurrence.

**Clinical discussion:**

The recommended follow-up of colorectal cancer patients is 5 years. However, there are rare instances of late metachronous liver recurrences, that suggest the necessity of more continuous surveillance. For patients with CRLM surgical resection is considered the most radical treatment of choice. The anatomy of Laennec's capsule allows the precise isolation of hepatic pedicles and can facilitate anatomical hepatectomy.

**Conclusion:**

We present a rare case report of borderline-resectable CRLM successfully treated with central liver resection with application of Laennec's approach a decade after the resection of a primary colorectal tumor.

## Highlights

• There are rare cases of colorectal cancer liver metastasis occurred after the termination of standard 5-year follow-up
• Radical resection is feasible in patients with metastatic lesion adjacent to hepatic hilum after preoperative chemotherapy
• Surgical approach based on the anatomy of the Laennec’s capsule facilitates anatomical liver resection

## 1 Introduction

Colorectal cancer (CRC) is the 3rd most common cancer type worldwide and the 4th in Ukraine [1,2]. CRC cells have the potential to disseminate from their origin in the colon to other areas of the body, often before the primary tumor is detectable [3]. It was shown that as few as 10^5^ cells (∼0.001 cm^3^) can initiate the CRC metastatic process [4]. A fraction of CRC cells might carry distinct biological traits conferring metastatic potential. Concurrently, dormant tumor cells have the ability to survive in the specific sites such as liver, lungs, bone marrow, lymph nodes, potentially reactivating after years of successful treatment of the primary tumor to initiate a metastatic disease [5].

Current treatment and surveillance guidelines recommend chest/abdominal/pelvic computer tomography (CT) and colonoscopy from the date of surgery for a total of 5 years for stage II-IV CRC [6]. However, 7–11 % of patients may develop a late recurrences after 5 years from initial colorectal surgery [7]. Therefore, the significance of extended monitoring beyond the initial 5 years remains a crucial inquiry.

For the last 3 decades, radical resection of the primary colorectal tumor and metastatic lesions provides the best oncological results and remains the standard of treatment [8]. There are various surgical techniques that facilitate liver resection. Extrahepatic Glissonean isolation based on the anatomy of Laennec's capsule allows precise isolation of segmental hepatic pedicles, and can be used for anatomical hepatectomy [9,10].

In this case report we present our experience of applying Laennec membrane-tunnel approach to treat a patient with delayed onset of borderline-resectable CRLM. Our work has been written according to the SCARE Guidelines 2020 criteria [11].

## 2 Case presentation

**Fig. 1:**
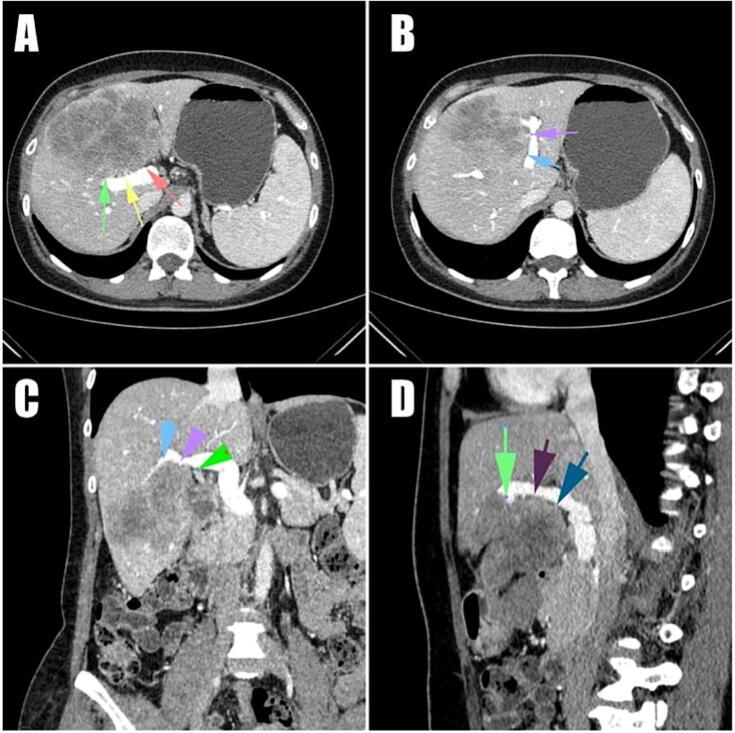
CT scans before the CTx.

A 38-year-old women complained of the pain in the upper right abdominal quadrant was referred to our hospital. 10 years before the current episode, the patient underwent surgery due to the sigmoid colon adenocarcinoma: pT3pN0cM0 LV0 Pn0 R0 IIA stage, MSS/pMMR [12]. She was negative for Lynch syndrome germline genetic testing. The latest follow-up, that was conducted 5 years prior to the current visit, revealed a disease-free status. Current chest/abdominal/pelvic CT showed the mass in Sg4 and Sg5 of the liver, spread to the ventral part of Sg8, 126x86mm in size (Fig. 1).

The tumor mass in 90 % contact with the left portal vein (LPV), and the involvement of the Sg4a and Sg4b Glissonian pedicles (B). The right portal vein (RPV) and the right anterior portal vein (RAPV) were in contact up to 90 %, the ventral branch of Sg8 was within the thickness of the tumor mass (A, C, D).

After the radiological visualization and core needle biopsy the patient was diagnosed with the borderline-resectable solitary late metachronous colorectal adenocarcinoma liver metastasis pMMR/MSS, KRAS, NRAS and BRAF wild-type. Multidisciplinary team recommended FOLFOX + cetuximab regimen with re-evaluation for conversion to resectable every 3 chemotherapy (CT) cycles. On the 3rd month of radiological follow-up, after 6 cycles of CT, a 13 % decrease of liver lesion was registered, which corresponded to the stabilization of the disease [13].

**Fig. 2:**
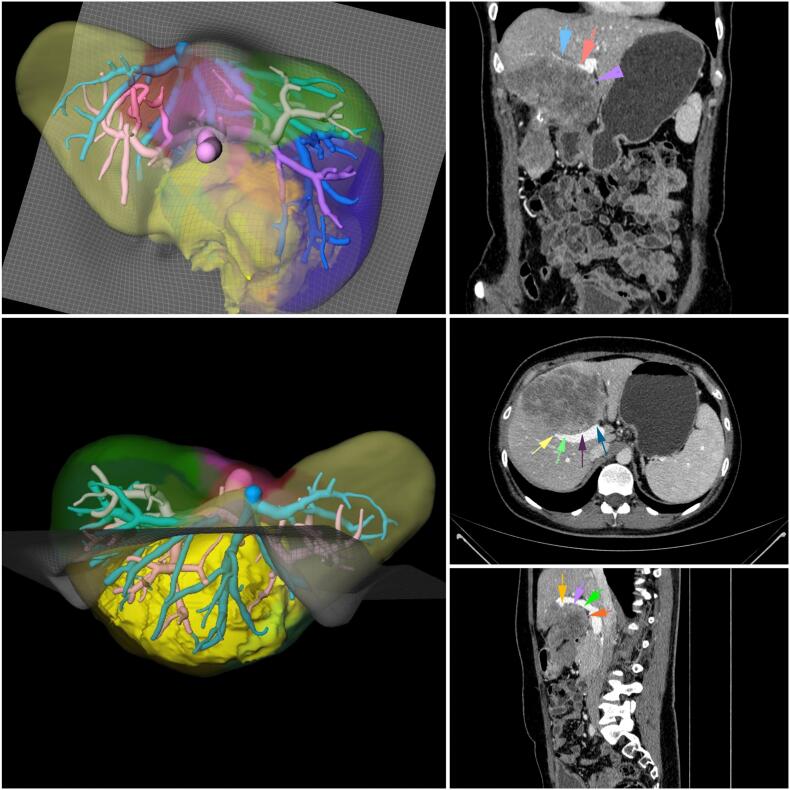
2D and 3D CT imaging of the patient after 3 months of CH.

According to CT and MRI data, there was no reliable evidence of tumor invasion to the hepatic hilum (Fig. 2). The decision was made to perform a central liver resection (Sg4, Sg5, Sg8v + middle hepatic vein) with surgical skeletonization of 1st and 2nd order hepatic pedicles.

### 2.1 Surgical technique

**Fig. 3:**
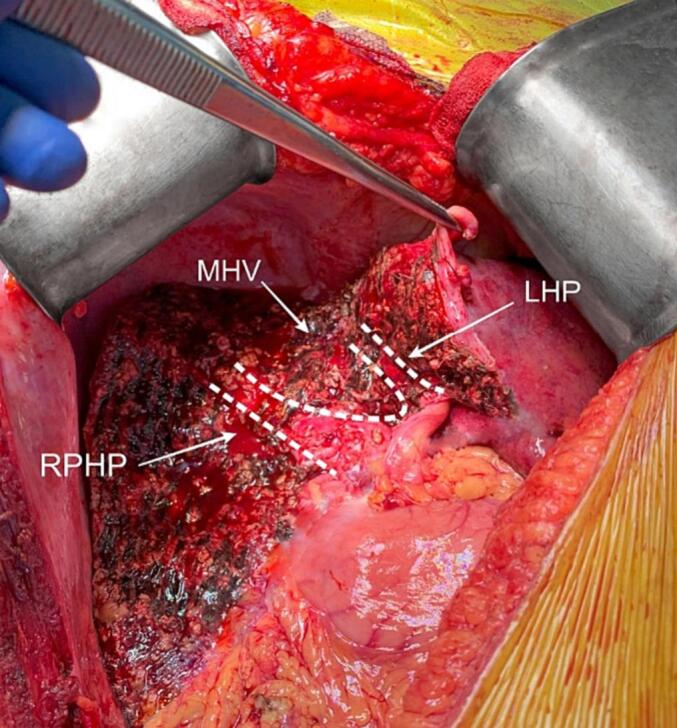
Photograph of the surgical field after the completion of resection. RPHP: right posterior hepatic pedicle. LHP: left hepatic pedicle. MHV: middle hepatic vein stump.

The J-shaped surgical incision was used. We conducted intraoperative ultrasonography to get a precise understanding of the location of metastatic mass and its relation to the hepatic Glissonian pedicles. Liver mobilization included dissection of the falciform and round ligaments with preservation of the left and right triangular ligaments. After lowering of the hilar plate, Laennec's membrane-tunnel approach was applied to isolate Glissoneal pedicles [9,10]. Liver parenchyma transection was begun from the right side through the right portal scissure with anatomical resection of Sg5 and to the RAPV with subsequent surgical skeletonization of the right hepatic pedicle. Further parenchymal transection from the left side of the lesion followed the umbilical scissure, with transection within the gap between Laennec's capsule and liver hilar plate, along the left hepatic pedicle. After two transection lines joined in one, the confluence of left and right portal pedicles was detached from the metastatic mass. 3rd order pedicles to Sg4 and Sg5, and to the ventral portion of the Sg8 were ligated within the Laennec's gap, and parenchymal transection was continued through the umbilical scissure. Middle hepatic vein was mobilized and transected with Prolene 4.0 suturing. Further parenchymal transection between paracaval portion of Sg1 and anterior section + Sg4 allowed to complete the procedure (Fig. 3).

Liver transection was conducted using crash-clamp technique and application of the intermitted Pringle maneuver (20 min ischemia, 5 min reperfusion). Blood loss was 350 ml, blood components transfusion wasn't performed. Total surgery duration was 310 min with 65 min of liver warm ischemia. Postoperative period was uneventful and patient was discharged on the 5th postoperative day. Histological report of the resected specimen revealed metastasis of intestinal adenocarcinoma G2 with large focal areas of necrosis, surgical margins were described as R1vasc for the perihilar area and R0 for the parenchymal margin.

### 2.2 Follow up

**Fig. 4:**
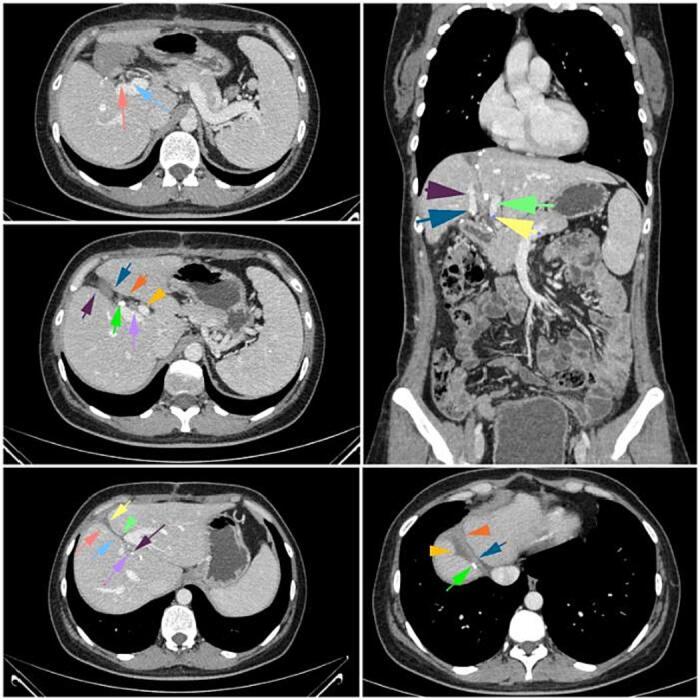
CT imaging at the 18th month after the surgery.

The surveillance chest/abdominal/pelvic CT and regular monitoring of carcinoembryonic antigen blood levels were conducted at 1, 3, 6, 9, 12, 15 and 18 months after surgery, revealing no evidence of local or distant metastatic recurrence (Fig. 4).

## 3 Discussion

In 2020, the global incidence of colorectal cancer reached nearly two million cases, 16,700 new patients were registered in Ukraine [1,2]. Liver represents the predominant site of colorectal cancer distant metastasis. Approximately half of all patients develop liver metastasis during the course of their disease [14]. For the past three decades surgical resection remains the only treatment modality with curative intent [8]. The evolution of metastatic CRC treatment is an example of continuous advancements within the oncological field. The pioneers of hepatopancreatobiliary surgery used conservative technologies in the treatment of primary tumor and liver metastasis, due to limited knowledge in the anatomy and physiology of the organ and the low effectiveness of chemotherapy of that time. At the end of the 1980s, liver resection was not recommended for patients with more than four CRLM, since their life expectancy did not exceed 3 years [15]. However, the growing adoption of aggressive surgical approaches has expanded the indications for resection.

Intraoperative ultrasound made it possible to remove metastasis located deeply in the parenchyma in an amount of up to 50 lesions during single surgical procedure [16]. Further understanding of the organ function, its anatomy, the metastatic disease biology stimulated surgeons and scientists to explore safer surgical techniques for patients with complex liver lesions. The organ's unique capacity to regenerate has led to development of two-stage liver resections for the patients who were previously considered inoperable [17]. The group of surgeons introduced the technique of one-stage parenchymal sparing liver resections. That implied the tumors' detachment from intrahepatic vascular structures, blood flow control and identification of communicant vessels among hepatic veins, following the hepatic vein clamping [18].

Furthermore, the updated understanding of the liver's surgical anatomy, including the anatomy of Laennec's capsule, has contributed to the comprehension of the R1 vascular margin safety phenomenon [19]. Indeed, Laennec's capsule was first described in 1802, as a membrane, that lays over the liver. Almost 200 years later it was revealed that it covers not only the capsule but also surrounds the Glissonean pedicles and the liver plate system [9]. There's a gap between Laennec's capsule and hepatic pedicles, that permits the extrahepatic isolation of Glissonean pedicles and can be used as an anatomical landmark to expose the hepatic pedicles and perform the tumor detachment from vascular structures, as described in current case.

## 4 Conclusion

We describe a successful case of a surgical treatment of a borderline-resectable metastatic lesion adjacent to the portal bifurcation in the hepatic hilum. The tumor was removed with 180 degree left and right Glissonean pedicles skeletonization within the Laennec's capsule gap. Disease-free and recurrence-free 18 months postoperative follow-up demonstrates good oncological result. Laennec's approach provides new possibilities for safe liver resection for centrally located colorectal metastatic lesions.

## Consent

Written informed consent was obtained from the patient for publication of this case report and accompanying images. A copy of the written consent is available for review by the Editor-in-Chief of this journal on request.

## Ethical approval

According to our institutional policy, retrospective studies do not require ethical committee approval.

## Funding

This research did not receive any specific grant from funding agencies in the public, commercial, or not-for-profit sectors.

## Author contribution

Anton Burlaka, Andriy Beznosenko– study concept and writing the initial draft.

Veronika Rozhkova – data collection, editing and submitting the manuscript.

Romanna Pavliuk, Oleksandr Chukanov– radiological images design.

All authors took part in final revision and preparation of the manuscript.

## Guarantor

Veronika Rozhkova.

## Research registration number

Our paper does not describe novel intervention in humans, hence, isn’t qualified as First in Man case report.

## Conflict of interest statement

Authors declare no competing interests.
